# Efficacy and Safety of Combined Catheter Ablation and Left Atrial Appendage Closure in Atrial Fibrillation: A Systematic Review and Meta-Analysis

**DOI:** 10.7759/cureus.82817

**Published:** 2025-04-23

**Authors:** Anurag Rawat, Syed Ali Ahsan, Sanjay Eda, Abdallah A Riyalat, Heer M Joshi, Sandipkumar S Chaudhari, Calvin R Wei, Neelum Ali

**Affiliations:** 1 Interventional Cardiology, Himalayan Institute of Medical Sciences, Dehradun, IND; 2 Medicine, King Edward Medical University, Lahore, PAK; 3 Medicine, MNR Medical College and Hospital, Fasalwadi, IND; 4 Pediatric Medicine, Sidra Medicine, Doha, QAT; 5 Internal Medicine, Jackson Park Hospital, Chicago, USA; 6 Cardiothoracic Surgery, University of Alabama, Birmingham, USA; 7 Family Medicine, School of Medicine and Health Sciences, University of North Dakota, Fargo, USA; 8 Research and Development, Shing Huei Group, Taipei, TWN; 9 Internal Medicine, University of Health Sciences, Lahore, PAK

**Keywords:** atrial fibrillation, catheter ablation, left atrial appendage closure, meta-analysis, stroke prevention

## Abstract

The combination of catheter ablation (CA) and left atrial appendage closure (LAAC) has emerged as a potential therapeutic strategy for patients with atrial fibrillation (AF). This systematic review and meta-analysis evaluated the efficacy and safety of the combined approach compared with CA alone. We conducted a comprehensive search of PubMed, Embase, Web of Science, and the Cochrane Library from inception to January 10, 2025. Studies comparing CA plus LAAC with CA alone were included. Of 1,066 identified articles, 11 studies met the inclusion criteria. The mean follow-up duration ranged up to 24 months, with reported CHA₂DS₂-VASc scores ranging from 2.2 to 4.3 and HAS-BLED scores from 2.0 to 3.7. Meta-analysis showed no significant difference in thromboembolic events between the combined and control groups (RR: 1.42, 95% CI: 1.10-1.83, I² = 0%), and no significant difference in arrhythmia recurrence (RR: 1.02, 95% CI: 0.84-1.24, I² = 28%). However, the combined approach was associated with a significantly higher risk of procedural complications (RR: 1.61, 95% CI: 1.01-2.59, I² = 0%). These findings suggest that adding LAAC to CA does not reduce thromboembolic events or arrhythmia recurrence but may increase the risk of procedural complications. Given the predominance of observational studies and limited randomized controlled trial (RCT) data, larger high-quality trials are needed to more definitively assess the role of combined CA and LAAC in AF management.

## Introduction and background

Atrial fibrillation (AF) is the most common sustained cardiac arrhythmia, affecting millions worldwide and contributing significantly to morbidity and mortality [1,2]. Its clinical consequences include an increased risk of stroke, systemic embolism, heart failure, and impaired quality of life [2]. The management of AF is multifaceted, encompassing strategies for rate control, rhythm control, and thromboembolic risk reduction [3]. Among these, catheter ablation (CA) has emerged as a cornerstone in rhythm control for patients with symptomatic AF refractory to or intolerant of antiarrhythmic drugs [4,5]. By targeting ectopic foci and modifying atrial substrate, CA aims to restore and maintain sinus rhythm, thereby improving symptoms and cardiac function [6]. However, despite its efficacy, CA alone does not eliminate the thromboembolic risk associated with AF, particularly in patients with a high CHA₂DS₂-VASc score. 

Simultaneously, percutaneous left atrial appendage closure (LAAC) has gained recognition as a viable alternative for stroke prevention in AF patients who are unsuitable for long-term oral anticoagulation (OAC) [7]. The left atrial appendage (LAA) is the predominant site of thrombus formation in non-valvular AF, and its occlusion has demonstrated efficacy in reducing stroke risk while mitigating bleeding complications associated with OAC [8]. Devices such as the Watchman and Amulet have shown promising results in clinical trials and real-world studies, paving the way for LAAC to be incorporated into clinical practice guidelines [9]. 

Combining CA and LAAC in a single procedural approach represents an innovative strategy to address both rhythm control and stroke prevention in AF patients, particularly those with high thromboembolic and bleeding risks [10]. The synergistic benefits of this combined approach lie in its potential to achieve arrhythmia suppression through ablation while simultaneously mitigating thromboembolic risk via appendage closure [11]. This dual therapy approach is especially appealing to patients with complex clinical profiles, including those with contraindications to OAC or recurrent AF after initial ablation [12]. However, the integration of CA and LAAC in routine clinical practice raises important questions regarding its efficacy, safety, and cost-effectiveness compared to standalone strategies [13]. Procedural considerations, such as the timing and sequence of interventions, device selection, and operator expertise, also play a pivotal role in determining outcomes [12]. Moreover, long-term data on stroke prevention, arrhythmia recurrence, and quality of life are essential to substantiate the benefits of this combined approach. 

This systematic review and meta-analysis aim to comprehensively evaluate the efficacy and safety of CA combined with LAAC in treating AF. By synthesizing data from randomized controlled trials (RCTs) and observational studies, this analysis seeks to provide evidence-based insights into the clinical utility of this novel strategy, guiding clinicians in optimizing management for AF patients with complex therapeutic needs. 

## Review

Methodology 

Literature Search 

 A comprehensive literature search was conducted across multiple electronic databases, including PubMed, Embase, Web of Science, and the Cochrane Library from the inception of databases to 10 January 2025. The search strategy was designed to identify studies evaluating the efficacy and safety of CA combined with LAAC in patients with AF. Keywords and Medical Subject Headings (MeSH) terms related to "catheter ablation," "left atrial appendage closure," "atrial fibrillation," “combination,” "stroke prevention," and "thromboembolic risk" were used. No language restrictions were applied. Bibliographies of relevant articles were also screened for additional eligible studies. 

Study Selection 

Studies were included if they met the following criteria: RCTs or observational studies evaluating the combined use of CA and LAAC in adult AF patients; reporting outcomes such as death, thromboembolic events, bleeding events, and peri-procedural complications; and providing sufficient data for extraction. Studies focusing solely on CA or LAAC without combining both interventions were excluded. Studies lacking control or comparison groups were also excluded. Additionally, we excluded reviews, case reports, case series, animal studies, and editorials. Two independent reviewers screened titles and abstracts for eligibility, followed by a full-text review for final inclusion. Any disagreement between the two authors was resolved through discussion.

Data Extraction 

Data were extracted independently by two reviewers using a standardized form developed using Microsoft Excel (Microsoft Corp, Redmond, WA). The form was initially reviewed by two authors before approving it for use. Extracted data included study characteristics (author, year, design, and sample size), patient demographics, and reported outcomes. The outcomes assessed were death, thromboembolic events, bleeding events, and peri-procedural complications. Any discrepancies in data extraction were resolved through discussion or, if necessary, consultation with a third reviewer.

Quality Assessment 

The quality of included studies was assessed using the Cochrane Risk of Bias tool for RCTs and the Newcastle-Ottawa Scale for observational studies. Assessment criteria included selection bias, performance bias, detection bias, and reporting bias for RCTs, as well as selection of participants, comparability of study groups, and ascertainment of outcomes for observational studies. Studies scoring 7-9 stars were considered to have good quality, those with 4-6 stars had a fair quality, and those scoring ≤3 stars were classified as having a low quality. Quality assessment was performed by two authors. Any disagreements between the two were resolved through discussion.

Data Analysis 

Data were synthesized quantitatively using meta-analytic techniques. Pooled estimates of outcomes were calculated using random-effects models to account for heterogeneity among studies. To compare outcomes between the two groups, the risk ratio (RR) was calculated along with 95% CI. A P-value less than 0.05 was considered significant. Heterogeneity was assessed using the I² statistic, with values >50% indicating substantial heterogeneity. Subgroup analyses were performed based on study design, patient characteristics, and procedural details. Publication bias was evaluated using funnel plots and Egger’s test. All statistical analyses were conducted using Review Manager version 5.4.1 (Cochrane Collaboration, London, England).

Results 

**Figure 1:**
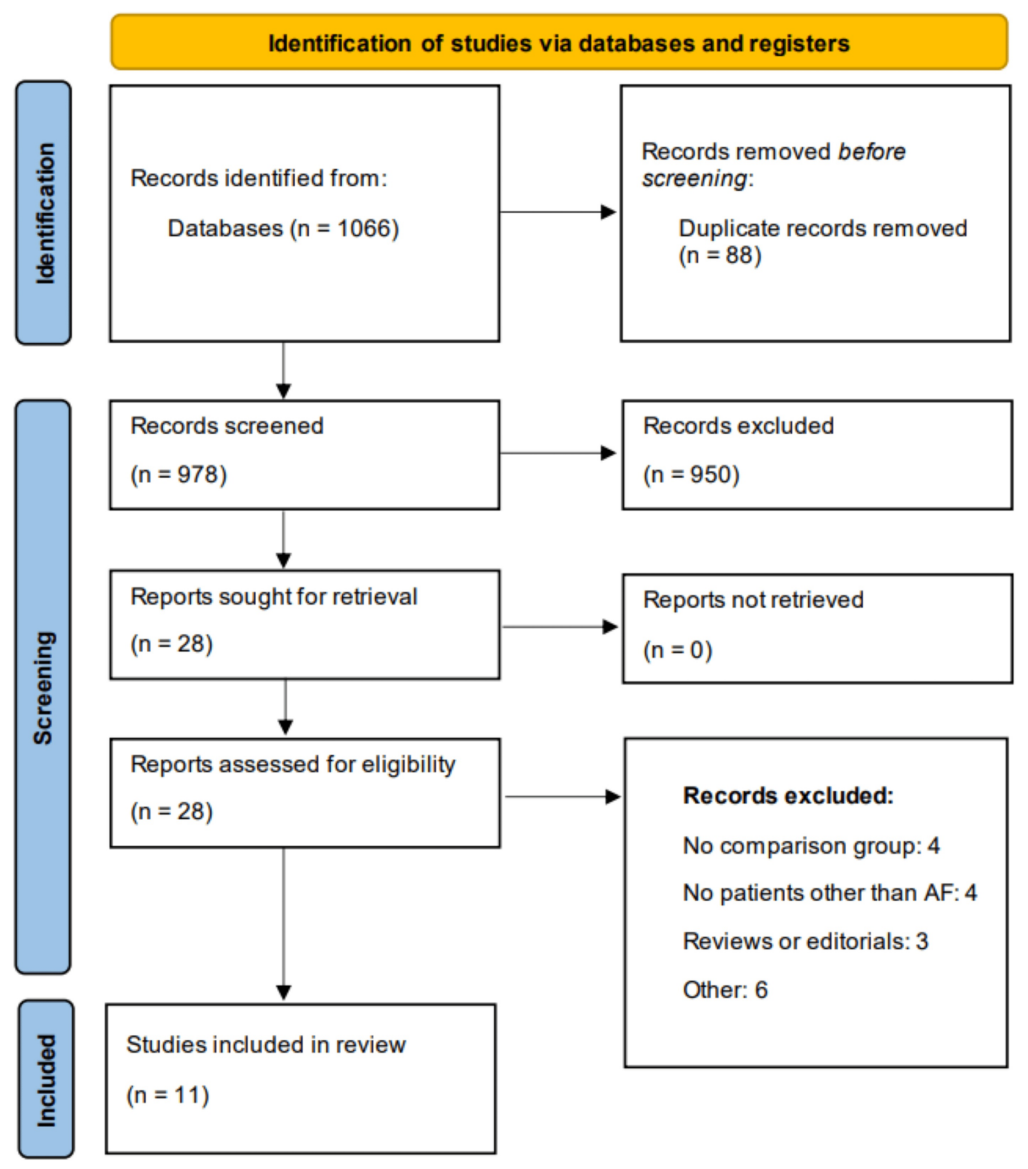
PRISMA flowchart of study selection

We identified 1,066 articles through online database searching. After removing duplicates, 978 articles were initially screened using their abstract or title based on pre-defined inclusion and exclusion criteria. We removed irrelevant or repetitive records and 28 articles were further screened in details using their full-text. Ultimately, 11 articles met the inclusion criteria and included in the meta-analysis. Figure 1 describes the study selection process. 

Characteristics of Included Studies 

Table 1 presents the characteristics of all included studies. A total of 38,432 participants were included in this meta-analysis including 19,239 subjects in CA plus LAAC group. The mean follow-up duration of included studies ranged up to 24 months. The average CHA2DS2-VASc score and HAS-BLED score ranged from 2.2 to 4.3 and 2.0 to 3.7, respectively. Table 2 presents a risk of bias assessment of included observational studies.

**Table 1:** Characteristics of included studies RCT, randomized control trial; NR, not reported

Author	Year	Study design	Region	Groups	Sample size	Follow-up	Age	Males	Diabetes	Hypertension	CHA2DS2-VASc score	HAS-BLED score
Fei et al. [14]	2023	Retrospective	China	Combined	252	12 months	NR	NR	NR	NR	NR	3.1
				Control	157							1.6
Mo et al. [15]	2020	Case-control	China	Combined	76	24 months	69.9	39	14	56	3.6	3.3
				Control	76		69.5	36	15	54	3.4	2.6
Panikker et al. [16]	2016	Retrospective	Singapore	Combined	20	12 months	68	13	NR	NR	3.1	2.5
				Control	40		67	26			3	2.3
Pelissero et al. [17]	2017	Retrospective	Italy	Combined	21	12 months	66.86	14	NR	14	2.8	3.2
				Control	21		68.42	15		15	2.01	3.1
Ren et al. [18]	2020	Retrospective	China	Combined	42	12 months	70	26	8	26	3.8	3.7
				Control	262		66.3	142	60	154	2.8	2.7
Romanov et al. [19]	2020	RCT	United States	Combined	45	24 months	60	28	9	38	2.2	3.5
				Control	44		60	26	11	33	2.3	3.4
Uwumiro et al. [20]	2024	Retrospective	United States	Combined	18195	In hospital	68.2	11827	5531	6186	NR	NR
				Control	18195		68.3	11827	5422	6350		
Wilber et al. [21]	2021	RCT	United States	Combined	404	12 months	66.2	288	75	332	NR	NR
				Control	206		67.4	158	48	174		
Yang et al. [22]	2021	Retrospective	China	Combined	65	12 months	61.8	40	9	45	3	3
				Control	65		60.7	43	10	50	4	3
Yang et al. [23]	2022	Retrospective	China	Combined	62	3 months	64.2	32	9	31	3.8	3
				Control	62		62.5	37	9	39	3.3	2
Zhu et al. [24]	2020	Retrospective	China	Combined	56	12 months	65.2	33	13	39	4.3	2
				Control	56		64.8	34	11	39	4.1	1.8

**Table 2:** Quality assessment of included studies (observational studies)

Author	Selection	Comparison	Assessment	Overall
Fei et al. [14]	4	2	3	Good
Mo et al. [15]	3	2	3	Good
Panikker et al. [16]	2	2	3	Good
Pelissero et al. [17]	3	1	2	Fair
Ren et al. [18]	4	2	2	Good
Uwumiro et al. [20]	4	2	3	Good
Yang et al. [22]	3	2	2	Good
Yang et al. [23]	2	2	2	Fair
Zhu et al. [24]	3	2	3	Good

Meta-Analysis of Outcomes 

**Figure 2:**
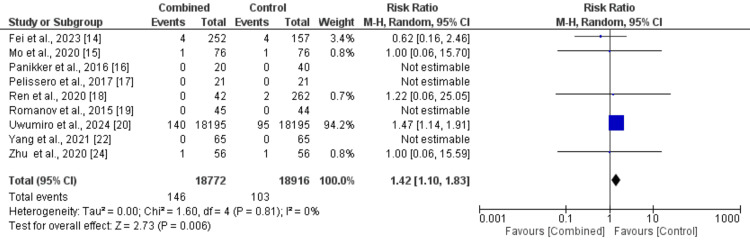
Comparison of treatment groups on risk of thromboembolic events

**Figure 3:**
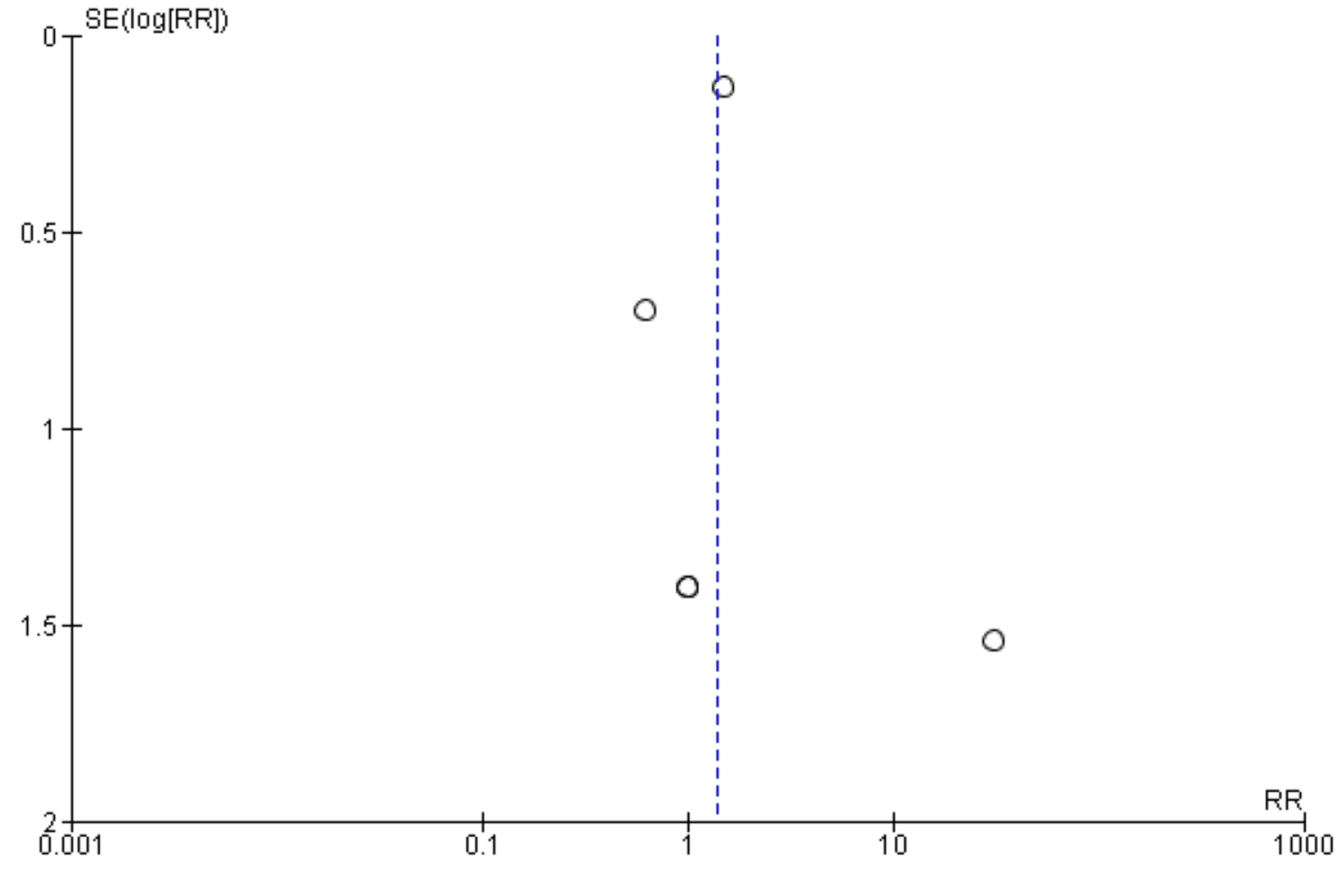
Funnel plot showing publication bias (thromboembolic events)

Thromboembolic events: We included nine studies in the pooled analysis comparing thromboembolic events between patients in the CA plus LAAC group and those in the ablation-only group. The results, presented in Figure 2, indicate a high risk of thromboembolic events in a combined group compared to the control group (RR: 1.42, 95% CI: 1.10-1.83). Moderate heterogeneity was observed among the study results (I² = 0%). Figure 3 assessed there was evidence of publication bias in the funnel plot analysis, with asymmetrical distribution of studies around RR = 1 as most included studies have null values that are not represented in the funnel plot. A study performed by Uwumiro et al. [20] assessed events only during a stay in the hospital. We performed sensitivity analysis by removing the study and found that there is still no significant difference between the two groups in terms of thromboembolic events (RR: 0.78, 95% CI: 0.27 to 2.22, I-Square: 0%). This study carried the majority of weight in the analysis due to the large sample size.

**Figure 4:**
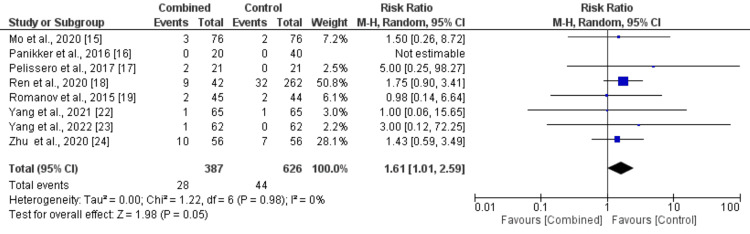
Comparison of treatment groups on the risk of complications

**Figure 5:**
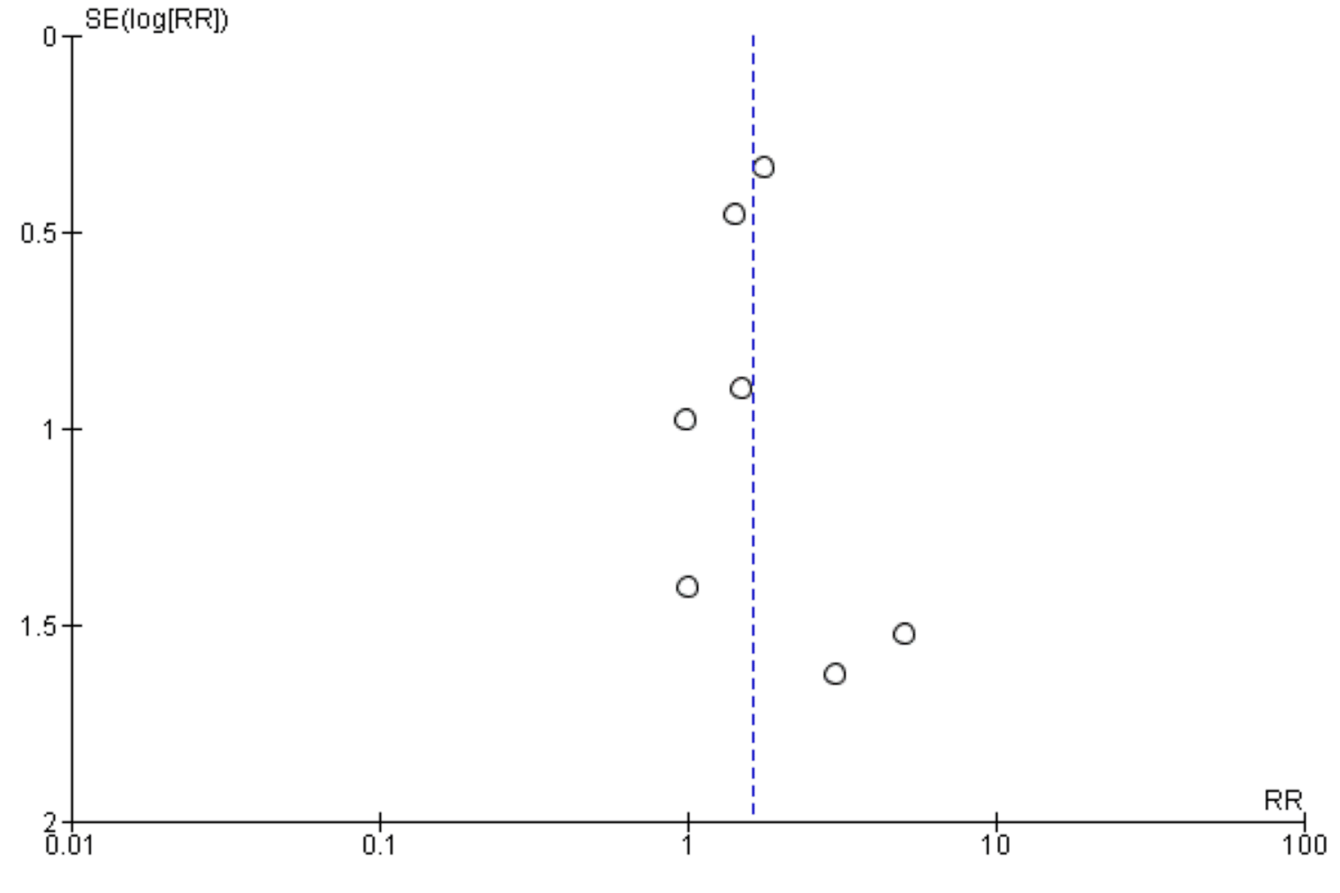
Funnel plot showing publication bias (procedural complications) RR, risk ratio

Procedural complications: Eight studies were included in the pooled analysis comparing procedural complications between patients in the CA plus LAAC group and the ablation-only group, with results presented in Figure 4. The analysis revealed a significantly higher risk of procedural complications in the CA plus LAAC group compared to the ablation-only group (RR: 1.61, 95% CI: 1.01-2.59). No heterogeneity was observed among the study results (I² = 0%). Figure 5 shows funnel plot analysis, which revealed potential publication bias with asymmetrical distribution of studies around the vertical line at RR = 1. The observed pattern shows clustering of studies on both sides of the null effect line, with smaller studies (higher SE values) predominantly reporting positive effects (RR > 1), suggesting possible selective reporting of findings.

**Figure 6:**
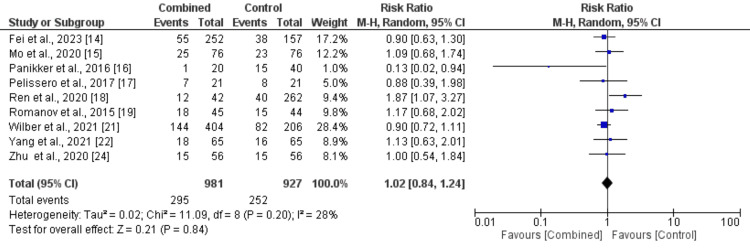
Comparison of treatment group in risk of arrhythmia recurrence

**Figure 7:**
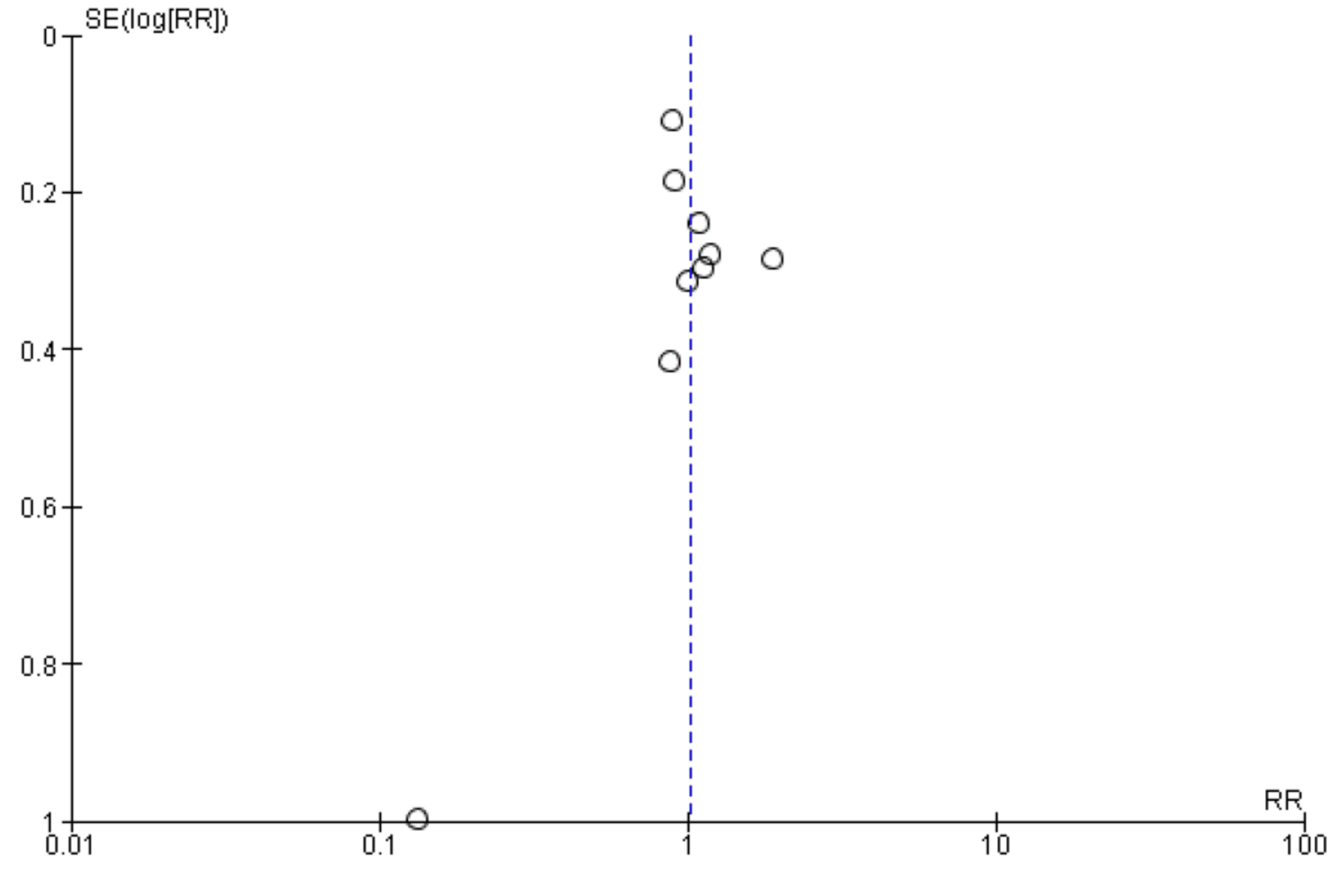
Funnel plot showing publication bias (arrhythmia recurrence)

Arrhythmia recurrence: Nine studies were included in the pooled analysis comparing arrhythmia recurrence between patients in the CA plus LAAC group and the ablation-only group, as presented in Figure 6. The analysis showed no significant difference in arrhythmia recurrence between the two groups (RR: 1.02, 95% CI: 0.84-1.24). No significant heterogeneity was observed among the study results (I² = 28%). Figure 7 showed a funnel plot, which demonstrated minimal evidence of publication bias, with studies distributed relatively symmetrically around the vertical reference line at RR = 1. Most studies clustered near the null effect line, with only one outlier showing a negative effect (RR ≈ 0.1), suggesting overall consistency in reported findings regardless of study precision.

Discussion 

This meta-analysis evaluates the effectiveness of the combined CA and LAAC strategy in patients with AF. The findings revealed no significant difference in arrhythmia recurrence between the two groups, but a higher risk of thromboembolic events and procedural complications was observed in the combined CA plus LAAC group compared to the ablation-only group.

Similarly, the meta-analysis by Junarta et al. [10] found no significant differences in the risk of arrhythmia recurrence or stroke/systemic embolism between the two groups. However, their findings on procedural complications differed from ours, as they reported no difference in complication rates. In contrast, a meta-analysis by Wang et al. [25] reported a higher complication rate in the combined group, consistent with our findings. Differences in study populations, operator experience, and procedural protocols may account for these discrepancies. Notably, the recruited patients in these studies were at moderate to high risk for bleeding events, as indicated by their HAS-BLED scores.

Our meta-analysis reported that thromboembolic events, including stroke and systemic embolism, were significantly higher in the CA plus LAAC group compared to CA alone. Generally, the stroke risk in patients with AF is significantly greater compared to patients without AF [26]. One crucial step in preventing thrombosis is anticoagulant therapy. However, certain patients are at high risk for stroke yet are not candidates for long-term anticoagulation. According to the 2020 ESC guidelines for the management of AF and the 2019 AHA/ACC/HRS guidelines, LAAC is a class IIb recommendation for such patients [27]. Nevertheless, our analysis suggests that the addition of LAAC may not reduce and may even increase the risk of thromboembolic events compared to CA alone. This finding challenges the presumed protective role of LAAC in patients undergoing ablation, particularly among those eligible for anticoagulation. Importantly, our meta-analysis did not address the discontinuation rates of anticoagulation therapy after the procedure, as none of the included studies reported long-term anticoagulation usage post-procedure. Future studies should investigate whether combining LAAC with CA safely facilitates anticoagulation discontinuation.

CA has been widely recommended in multiple guidelines as an effective treatment for AF. Recently, several studies explored combining CA with LAAC [14-24]. In the current meta-analysis, the combined strategy was associated with a higher risk of thromboembolic events, contrasting with previous findings such as the meta-analysis by Wang et al. [25], which found no significant advantage or disadvantage regarding thromboembolic events. Notably, our meta-analysis included two RCTs; one RCT reported no thromboembolic events in either group, while the other did not assess this outcome. Therefore, although our findings raise concerns, they are primarily derived from observational studies and should be interpreted with caution. Larger, high-quality RCTs are urgently needed to fully assess the efficacy of combining CA and LAAC for thromboembolic prevention.

A prior meta-analysis reported that LAA isolation could significantly reduce AF recurrence rates [28]. However, our analysis showed no significant difference in arrhythmia recurrence between the combined and ablation-only groups. This discrepancy may be due to the incomplete electrical isolation of the LAA during the LAAC procedure. Evidence from a dual approach combining LAA electrical isolation and CA showed higher freedom from AF at 12 months compared to CA alone (95% vs. 63%, P = 0.036) [16], although the study had limitations, including a small sample size and a non-randomized design. None of the studies in our analysis assessed procedural endpoints such as complete LAA isolation, which could explain the lack of effect on arrhythmia recurrence.

The higher complication rates observed in the CA plus LAAC group may be attributed to the combined procedural complexity [29]. Performing both procedures concurrently increases procedural duration and technical demands, which could raise the risk of complications like vascular injury, bleeding, or cardiac perforation [30]. Furthermore, LAAC involves deploying occlusion devices, which themselves carry risks such as device-related thrombus, embolization, or pericardial effusion [31]. The specific LAAC device types (e.g., Watchman vs. Amulet) were not stratified in our analysis. Future studies should explore whether device selection affects complication rates and procedural outcomes.

Additionally, procedural complications could be influenced by patient characteristics (e.g., CHA₂DS₂-VASc and HAS-BLED scores) or operator experience. It is plausible that higher-risk patients were selected for the combined procedure, potentially confounding the results. The included studies did not provide subgroup analyses based on procedural volumes or center experience. Outcomes might also improve over time with better operator proficiency and advances in procedural techniques.

It is also important to note that our findings are primarily based on non-randomized studies, except for two RCTs with limited reporting on thromboembolic outcomes. Therefore, the observed increased risk of thromboembolic events and procedural complications should be considered hypothesis-generating rather than definitive. Larger, well-designed RCTs with long-term follow-up are urgently needed to refine patient selection criteria and better understand the safety and efficacy of combining CA with LAAC in AF management.

Study Limitations 

The present study has several limitations. First, the number of included studies was limited, and the majority were observational in nature. The absence of RCTs makes it challenging to interpret the findings in a generalized context, as observational studies are more prone to biases and confounding factors like selection bias. Second, variations in the CA techniques and the types of occlusion devices used across the included studies may have introduced heterogeneity and potential bias in the results. Additionally, due to the lack of granular data from individual studies, we were unable to perform subgroup analyses to explore the impact of specific factors, such as patient characteristics, procedural variations, or device types, on the outcomes. Finally, our meta-analysis may be affected by publication bias, as evidenced by the asymmetry in the funnel plots because several studies reported no incident in outcomes potentially due to shorter follow-up duration. These limitations underscore the need for well-designed RCTs and standardized reporting in future research to provide more robust evidence. 

The findings of this study highlight several important directions for future research. First, the limited number of included studies and the predominance of observational designs emphasize the urgent need for well-designed RCTs to provide more robust evidence on the effectiveness and safety of combined CA and LAAC strategies. Second, the variability in ablation techniques and occlusion devices used across studies suggests the importance of standardizing procedural protocols to minimize heterogeneity and bias. Future studies should also focus on reporting granular data to enable subgroup analyses with longer follow-up duration, which could help identify specific patient populations or procedural factors associated with better outcomes. 

## Conclusions

Based on our meta-analysis of 11 studies including 38,432 participants, the combination of CA and LAAC showed no significant advantage over CA alone in preventing thromboembolic events or reducing arrhythmia recurrence in AF patients. However, the combined approach was associated with a higher risk of procedural complications. These findings suggest that careful patient selection and risk stratification are essential when considering this dual intervention strategy. Given the limitations of current evidence, primarily derived from observational studies, there is an urgent need for well-designed randomized controlled trials to definitively establish the role of combined CA and LAAC in AF management, particularly focusing on long-term outcomes and specific patient populations who might benefit most from this approach.
